# First Surveillance of Violence against Women during COVID-19 Lockdown: Experience from “Niguarda” Hospital in Milan, Italy

**DOI:** 10.3390/ijerph18073801

**Published:** 2021-04-06

**Authors:** Giulio Nittari, Getu Gamo Sagaro, Alessandro Feola, Mattia Scipioni, Giovanna Ricci, Ascanio Sirignano

**Affiliations:** 1 Telemedicine and Telepharmacy Center, University of Camerino, 9-62032 Camerino, Italy; getugamo.sagaro@unicam.it; 2 Department of Experimental Medicine, University of Campania “Luigi Vanvitelli”, Via Luciano Armanni, 5-80138 Naples, Italy; alessandro.feola@unicampania.it; 3 School of Law, University of Camerino, Via A. D’Accorso, 16-62032 Camerino, Italy; scipioni.mattia@outlook.it (M.S.); giovanna.ricci@unicam.it (G.R.); ascanio.sirignano@unicam.it (A.S.)

**Keywords:** violence, domestic violence, gender-based violence, covid-19, lockdown

## Abstract

Violence against women emerges with tragic regularity in the daily news. It is now an evident trace of a dramatic social problem, the characteristics of which are not attributable to certain economic, cultural, or religious conditions of the people involved but affect indiscriminately, in a unanimous way, our society. The study is a survey about the number of hospital admissions due to episodes attributable to violence against women, recorded by the Niguarda Hospital in Milan in the period 1 March–30 May from 2017 to 2020. This period, in 2020, corresponds to the coronavirus Lockdown in Italy. All the medical records of the Emergency department were reviewed, and the extracted data classified in order to identify the episodes of violence against women and the features of the reported injuries and the characteristics of the victims. The data did not show an increase in the number of cases in 2020 compared to previous years, but we did find a notable increase in the severity of injuries.

## 1. Introduction

Episodes of violence against women emerge with tragic regularity in the daily news and are now an evident trace of a dramatic social problem, the characteristics of which are not attributable to certain economic, cultural or religious conditions of the people involved, but affect without distinction, in a unanimous manner, our society [1]. Offenders do not fit into a predetermined identikit; they do not correspond to any specific type of personality or category. Violence against women has a thousand faces and a thousand forms, but two important aspects are almost constants of the phenomenon: (1) the fact that harmful and abusive behaviors almost always mature in emotional, family, and/or couple relational contexts; (2) the extreme difficulty of making them emerge, given the links between the perpetrator and the victim, because they are considered to be relevant to the private sphere and, in a certain sense, confidential and not to be disclosed (*Ministero dello Sviluppo Economico (MISE)*, 2020) [2]. Gender-based violence is a radical and universal social scourge, so much so that it is defined as “*gender genocide*” (*Ministero degli Affari Esteri e della Cooperazione Internazionale*, n.d.) [3]. Femicides represent the most extreme and evident gestures of the reality of oppression, inequality, abuse, violence, and a systematic violation of human rights. The expression “*gender violence*” indicates all forms of violence, including the most varied cases: psychological, physical, sexual, economic as well as persecutory acts (*stalking*). Physical violence is the most evident form of this crime, configuring various crime types such as beatings, personal injuries, trespassing, and kidnapping (art. 582 and 583 of the criminal code art. 605 c.p.) [4]. Such coercion is inflicted with any act intended to inflict harm or terrorize the victim. The current pandemic modified many fields of our daily life [5,6,7].

Violence against women is a human rights violation that significantly impacts victims, families, and the community. In general, intimate partner violence (IPV) is the most common and occurs in the family and in a couple that presupposes coexistence [8]. Currently, rates of violence against women are on the rise, and are a particular concern during COVID-19 because the pandemic affects almost every country globally and causes a significant negative impact on health, economic and social aspects [9]. To contain the COVID-19 pandemic and prevent transmission, lockdowns and social distancing are among public health measures. There are several reasons why violence against women may have increased during the COVID-19 pandemic than before the outbreak. Movement restriction, social isolation, and economic insecurity are among the reasons why women’s vulnerability to violence increases after the COVID-19 pandemic around the world, particularly domestic violence incidents. In general, violence against women is one of the critical public health problems globally. In 2010, 30% of women aged 15 and over experienced IPV worldwide [10], with 38.6% and 6.3%, respectively, of women and men killed by their intimate partner [11,12]. Currently, due to COVID-19, the lack of police investigations and the small number of comprehensive post-mortem examinations could hide crime cases. Hence, medicolegal issues, including death related to domestic violence in the COVID-19 era, could effectively identify the actors through forensic investigation [13,14,15]. In this sense, harmful and abusive behaviors almost always mature in emotional, family, and/or couple relational contexts. In this work, a statistical evaluation was carried out about the violent events that occurred during the COVID-19 emergency and the consequent lockdown through a comparison of the cases from 2017 to 2020 concerning the lockdown months. The present study sought to analyze whether the lockdown period that took place in Italy from March 2020 to May 2020 changed intentional personal injuries to women, both from a numerical and from a severity point of view. We analyzed each medical record relating to cases of domestic violence in the period between March and May of the years 2017, 2018, and 2019, taking care to respect anonymity in all cases. Subsequently, these data were compared with those of March–May 2020 for the number of injuries and their severity, in order to evaluate the trend during the lockdown.

## 2. Materials and Methods

We employed a retrospective cross sectional study and analyzed data with the help of the “PIESSE software” (PIESSE Software, Latina, Italy) used by the ASST Grande Ospedale Metropolitano Niguarda in Milan. The software has been in use by this hospital for more than 10 years and is also used by other healthcare facilities in the Lombardy region, Italy. To respect the patients’ privacy, the authors were unable to establish the exact country of origin of each patient; therefore, it has only been possible to distinguish the victims as “*Italian*” or “*foreign*”. The Niguarda Hospital’s catchment area in Milan is extremely varied, being one of the largest hospitals in Northern Italy.

### 2.1. Data Collection

Data recording through the PIESSE software (PIESSE Srl, Latina, Italy) takes place in the following way. The patient arrives at the emergency room (no distinction is made as to whether this occurs via the patient’s own means of transportation or in an emergency vehicle) and is identified through an identity document or valid health card. The patent will fill in a specific form relating to the PIESSE program, under a nurse’s guidance. In the case of an unidentifiable patient (for example, a patient in a state of unconsciousness), the patient is temporarily identified with the wording “NN” (in Italian “unidentified”, literally “not identified”), followed by a progressive serial number, pending a valid identification document. The software then collects the patient data.

### 2.2. Data Analysis

The data analysis was carried out with the Health Director of the ASST Grande Ospedale Metropolitano Niguarda’s authorization. The medical records of women who came to this hospital in March, April, and May of the years 2017, 2018, 2019, and 2020 were taken into consideration. The purpose was to evaluate the variations (in terms of frequency and severity) of cases of violence against women during the period in which society was forced to remain in homes due to the pandemic being in progress, compared to normal. The purpose of this work is to assess whether cohabitation has caused the mistreatment of women to vary. Anonymity and protection of sensitive data have been guaranteed thanks to the exclusive extrapolation of data relating to sex, age, nationality, prognosis, and diagnosis. The study consists of the analysis of the number of hospital admissions of female subjects who arrived at the ASST Grande Ospedale Metropolitano Niguarda in Milan, in the years 2017–2020 in the interval of dates contiguous to the coronavirus emergency lockdown in Italy, i.e., from 1 March to 31 May 2020. The diagnoses were classified according to 3 levels of increasing severity, based on the days of prognosis established by the doctor. This distinction is proposed based on the diagnosis and prognosis of the medical record analyzed.

(1) Aggressions, Beatings, Ecchymosis: this category includes hospital admissions without obvious symptoms (therefore, only a report of aggression), bruises and “light” signs of beatings, etc.
(2) Multiple bruises, wounds, bites: include obvious wounds and signs of aggression, sometimes accompanied by fractures of various sizes.
(3) Head injuries: includes head injuries of any extent.

We have, therefore, divided the cases as follows: (1) “mild” diagnosis, with a prognosis of fewer than 21 days; (2) “moderate” diagnosis, with a prognosis lasting between 21 and 40 days; (3) “severe” diagnosis, with a prognosis of more than 40 days. Data processing was performed using Microsoft Excel software.

## 3. Results

**Figure 1:**
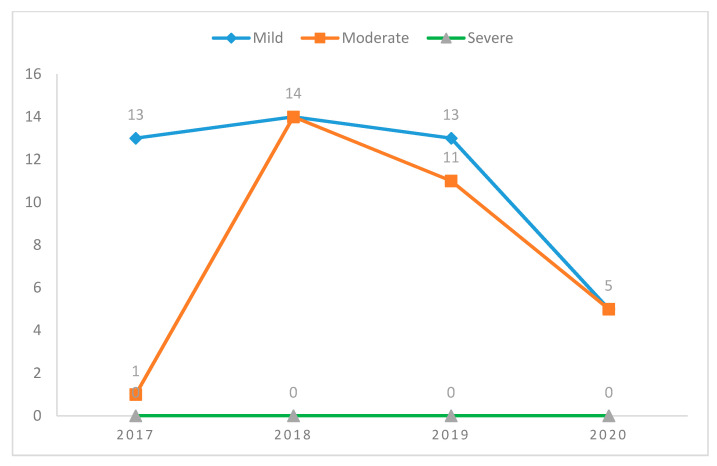
Time trend (2017–2020) of the number of cases of female violence that occurred over the years—in the periods corresponding to the lockdown (1 March–31 May of each year), divided according to their severity (mild, moderate or severe).

The data are shown in Figure 1, Table 1 and Table 2 relate to the number of cases registered in the period from 1 March to 31 May of each year, a period considered as “contiguous” to the Italian lockdown (officially started on 10 March and ended on 18 May 2020). Figure 1 shows the time trend of the number of female violence cases in the period 2017–2020, classified based on the prognosis.

No extremely severe cases (with a prognosis of more than 40 days) were recorded in the 4 years under study. Figure 1 shows a reduction in total violence cases, with zero severe, five mild, and five moderate cases. In 2020 there was a reduction in the total number of hospitalizations, with 10 cases, compared to 24 hospitalizations in 2019, 28 in 2018, and 14 in 2017 (Table 1). In addition, in 2020, an apparent aggravation of cases emerges, as the average duration of prognosis is greater than 21 days (against an average of 12–13 days in previous years) (Table 1). In all four years (except for 2018), the percentage of cases of violence suffered by foreign women was greater than or equal to that of Italian women.

## 4. Discussion

Violence Against Women (VAW) is a critical issue worldwide. There are several forms of VAW and there can be distinguished physical, sexual, psychological, economic abuse, and stalking, which constitute the five multi-faceted methods of violence and abuse that perpetrators utilize to achieve, maintain and regain control of their intimate partners or potential intimate partners [16]. To eradicate this phenomenon, the Council of Europe (CoE), in 2011, released a document, the so-called Istanbul Convention, on preventing and combating violence against women and domestic violence. The document is based on the understanding that violence against women is a form of gender-based violence that is committed against women because they are women. There are obligations that each signatory State should take to prevent violence against women, protect its victims and prosecute the perpetrators [17]. Italy ratified the Convention in 2012 and provided an effort with the Law n. 93 of 14 August 2013, which was then converted, with amendments, into Act n. 119 of 15 October 2013 “*New rules to combat gender-based violence which aim to prevent femicide and protect victims*”, which provides legal aid to victims, increased punishment in special cases, the possibility for the alleged victims of reporting their claims anonymously to the police, the introduction of a warning procedure in the case of stalking and, finally, the Law makes complaints non-revocable by the victim [16]. More recently, in 2019, the Italian Parliament introduced Law n.69 of 19 July 2019 (“Amendments to the Criminal Code, the Criminal Procedure Code and other provisions on the protection of victims of domestic and gender-based violence”), better known as the “Codice Rosso” Law. This latter law modifies the Italian Criminal Procedure Code regarding maltreatment in family, stalking and sexual violence, with the effect that any victim protection measures will be adopted more quickly. Besides, new crimes are introduced related to the unlawful dissemination of sexually explicit images or videos without the consent of the persons represented (“revenge porn”); deformation of the person’s appearance through permanent injuries to the face; coercion or induction into marriage; violation of the measures for removal from the family home; and the prohibition of approaching the places frequented by the victim of violence. Nevertheless, according to the WHO’s Global estimates, about 1 in 3 (35%) of women worldwide have experienced either physical and/or sexual intimate partner violence or non-partner sexual violence in their lifetime [18]. In 2014, the European Agency for Fundamental Rights (EUFRA) conducted a survey based on interviews with 42,000 women across the 28 Member States of the European Union (EU). The interviews’ focus was experiences of physical, sexual, and psychological violence, including incidents of intimate partner violence (“domestic violence”), and stalking, sexual harassment, and the role played by new technologies in women’s experiences of abuse [19]. According to the survey results, three women (33%) out of ten have experienced physical and/or sexual violence since the age of 15. One in five women (18%) has experienced stalking; every second woman (55%) has been confronted with one or more forms of sexual harassment. A similar survey was carried by the Italian Institute of Statistics (ISTAT) in 2014 and released in 2015 on a sample of 24,000 women aged 16–70 [20]. According to the ISTAT data, physical violence is more frequent among foreign women (25.7% vs. 19.6%), while sexual violence is more common among Italian women (21.5% vs. 16.2%). From our study, no significant difference emerged regarding the victim’s nationality, resulting in almost the same number of Italian and foreign patients in the months of 2020. The same result was observed in 2017, while in 2018, Italian women were more prevalent (53.57% vs. 46.43%); in 2019, foreign patients prevailed (33.33% vs. 66.67%). According to the ISTAT surveys, the age group most affected by physical violence episodes in the last 5 years was between 16 and 24 years, with 17.2% and 16.6%, respectively. It is interesting to highlight a reduction in sexual and physical violence cases (13.3% to 11.3%). However, it has been reported that the recent pandemic and the lockdown policies made by many countries caused an increase in violence against women both globally and in some EU-countries. Particularly, in France, the Interior Minister reported a 30% increase in domestic violence cases across the country and a 36% increase in Paris alone since 17 March 2020. In other EU-countries, an increasing number of emergency calls about domestic violence have been reported, with increases of 20% in Spain and 30% in Cyprus [21]. In this sense, the main risk of the lockdown policies, in terms of domestic violence, was the increase in the following risk factors: (1) quarantining the victim with an abusive partner, and (2) disconnecting the victim from their usual support system (family, friends, and community), with the consequent amplification of the difficulties in calling for help [21,22]. In addition, it has been hypothesized that isolation paired with psychological and economic stressors (e.g., unemployment, reduced income, limited resources, and limited social support) that accompany the pandemic, as well as potential increases in negative coping mechanisms (e.g., excessive alcohol consumption), can come together in a perfect storm to trigger violent behavior in the family [23]. The same risk factors for domestic violence were highlighted in other cases of natural disasters. Particularly, there have been increased domestic violence cases in Othello, Washington after the eruption of Mount St. Helens and after the storm of Hurricane Katrina [23]. The same trend has been reported following earthquakes, tsunamis, hurricanes, and many other catastrophic events worldwide, including the 2009 “Black Saturday” bushfires in Australia and the 2010 7.0 magnitude earthquake in Haiti [23,24]. Concerning the consequences of the violence, none of the cases considered in our study had a fatal outcome. However, it can be highlighted that the mean prognosis (expressed in days) has increased nearly twofold considering the cases from 2017 to the present. In the literature, case series of fatal VAW in Italy before the COVID-19 pandemic are reported [25,26,27]. This work presents some limitations. Particularly, the information is obtained only from the Emergency Department’s medical records, so there is no information on the continuation of the period of illness (any complications, etc.), causes or factors of the phenomena, or detailed information on the victim and attacker. Besides, this study is a retrospective case study and limited to the record or dataset’s variables.

## 5. Conclusions

The results of our study demonstrate that the total number of hospitalizations due to violence against women decreased from March to May 2020 compared to the same months in 2019, 2018, and 2017. However, violence against women still happens in our society, despite national and international regulatory efforts. Statistical studies that can map the phenomenon are also useful for identifying the critical points of the protection system of the subjects most at risk. Violence is more and more often conducted within the home, by husbands and relatives. The months of lockdown have also taken away the possibility of the abused woman to escape from the home by turning the house into a sort of prison [28]. From our investigation, it emerged that the women went to the hospital only when the violence became serious and the injuries could not be dealt with independently. Following our analysis, we could suggest a series of possible solutions to the problem, starting from—as an example—a survey at the municipal, regional, and provincial levels to monitor already known cases of domestic violence, in order to prevent further damage due to forced coexistence as a result of COVID-19 [29]. In cases of real danger, it is necessary to ensure the possibility that, after performing a swab to avoid contagion from COVID-19, women victims can be placed in a shelter or a similar structure.

## Figures and Tables

**Table 1:** Statistical data relating to cases of violence against women found in the medical records analyzed.

Year	N° Hospitalization	Average Age of Patients	N(%) Italian Patients; N(%) Foreign Patients	Average Prognosis Duration
2017	14	39.07	7 (50%); 7 (50%)	11.07 days
2018	28	36.68	15 (53.57%); 13(46.43%)	15.16 days
2019	24	35.83	8 (33.33%); 16 (66.67%)	12.70 days
2020	10	47.50	5 (50%); 5 (50%)	21.75 days

In all four years (except for 2018), the percentage of cases of violence suffered by foreign women was greater than or equal to that of Italian women.

**Table 2:** Number of cases of violence against women classified based on the diagnosis. Both the number of cases and the percentage of the total for each year are reported.

Diagnosis	2017 N Cases (%)	2018 N Cases (%)	2019 N Cases (%)	2020 N Cases (%)	Total N Cases (%)
Attacks, Beatings, Ecchymosis	4 (28.56%)	7 (25%)	8 (33.33%)	2 (20%)	21 (15.96%)
Multiple bruises, injured, Fractures	7 (50%)	17 (60.71%)	13 (54.17%)	6 (60%)	43 (56.58%)
Head trauma	3 (21.44%)	4 (14.29%)	3 (12.5%)	2 (20%)	12 (27.46%)
All diagnoses	14	28	24	10	76

## Data Availability

Data are available on request from the corresponding author.

## References

[1] Potenza S., Carella V., Feola A., Marsella L.T., Marella G.L. (2021). Femicide in a central Italy district (Southern Latium) in the period 1998–2018. Minerva Psichiatr..

[2] Ministero dello Sviluppo Economico (MISE) Femminicidio. https://www.mise.gov.it/images/stories/documenti/FEMMINICIDIO_per_web.pdf.

[3] Ministero degli Affari Esteri e della Cooperazione Internazionale Allegato 2. https://www.esteri.it/mae/approfondimenti/20090827_allegato2_it.pdf.

[4] Acquadro Maran D., Varetto A., Corona I., Tirassa M. (2020). Characteristics of the stalking campaign: Consequences and coping strategies for men and women that report their victimization to police. PLoS ONE.

[5] Ricci G., Pallotta G., Sirignano A., Amenta F., Nittari G. (2020). Consequences of COVID-19 Outbreak in Italy: Medical responsibilities and governmental measures. Front. Public Health.

[6] Bolcato M., Aurilio M.T., Aprile A., Di Mizio G., Della Pietra B., Feola A. (2021). Take-home messages from the COVID-19 pandemic: Strengths and pitfalls of the italian national health service from a medico-legal point of view. Healthcare.

[7] Ricci G., Campanozzi L.L., Nittari G., Sirignano A. (2020). Telemedicine as a concrete response to covids-19 pandemic. [La telemedicina come una risposta concreta alla pandemia da sars-cov-2]. Riv. Ital. Med. Leg. Dirit. Campo Sanit..

[8] Yahya A.S., Khawaja S., Chukwuma J. (2020). Association of COVID-19 with Intimate Partner Violence. Prim Care Companion CNS Disord [Internet]. https://www.psychiatrist.com/pcc/covid-19/intimate-partner-violence-and-covid.

[9] Peterman A., O’Donnell M. (2020). COVID-19 and Violence against womEn and Children: A Second Research Round Up. Cent Glob Dev [Internet]. https://www.cgdev.org/sites/default/files/covid-19-and-violence-against-women-and-children-second-research-round.pdf.

[10] Devries K.M., Mak J.Y.T., García-Moreno C., Petzold M., Child J.C., Falder G., Lim S., Bacchus L.J., Engell R.E., Rosenfeld L. (2013). The global prevalence of intimate partner violence against women. Science.

[11] Stöckl H., Devries K., Rotstein A., Abrahams N., Campbell J., Watts C., Moreno C.G. (2013). The global prevalence of intimate partner homicide: A systematic review. Lancet.

[12] World Health Organization (2013). Global and Regional Estimates of Violence against Women: Prevalence and Health Effects of Intimate Partner Violence and Non-Partner Sexual Violence [Internet]. www.who.int/about/licensing/copyright_form/en/index.html.

[13] Bogdanović M., Atanasijević T., Popović V., Mihailović Z., Radnić B., Durmić T. (2020). Is the role of forensic medicine in the covid-19 pandemic underestimated?. Forensic Sci. Med. Pathol..

[14] Roux C., Weyermann C. (2020). Can forensic science learn from the COVID-19 crisis?. Forensic Sci. Int..

[15] Barranco R., Ventura F. (2020). The role of forensic pathologists in coronavirus disease 2019 infection: The importance of an interdisciplinary research. Med. Sci. Law..

[16] Zara G., Gino S. (2018). Intimate partner violence and its escalation into femicide. Frailty thy name is “Violence against Women”. Front. Psychol..

[17] Council of Europe Council of Europe Convention on Preventing and Combating Violence against Women and Domestic Violence. https://www.coe.int/fr/web/conventions/full-list/-/conventions/rms/090000168008482e.

[18] World Health Organization Violence against Women. https://www.who.int/news-room/fact-sheets/detail/violence-against-women.

[19] European Union Agency for Fundamental Rights Violence against Women: An EU-Wide Survey. https://fra.europa.eu/sites/default/files/fra_uploads/fra-2014-vaw-survey-main-results-apr14_en.pdf.

[20] ISTAT La Violenza Contro le Donne Dentro e Fuori la Famiglia. 2020. https://www.istat.it/it/archivio/161716.

[21] Ertan D., El-Hage W., Thierrée S., Javelot H., Hingray C. (2020). COVID-19: Urgency for distancing from domestic violence. Eur. J. Psychotraumatol..

[22] Usher K., Bhullar N., Durkin J., Gyamfi N., Jackson D. (2020). Family violence and COVID-19: Increased vulnerability and reduced options for support. Int. J. Ment. Health Nurs..

[23] Campbell A.M. (2020). An increasing risk of family violence during the Covid-19 pandemic: Strengthening community collaborations to save lives. Forensic Sci. Int. Rep..

[24] Parkinson D. (2019). Investigating the increase in domestic violence post disaster: An australian case study. J. Interpers. Violence.

[25] Moreschi C., Da Broi U., Zamai V., Palese F. (2016). Medico legal and epidemiological aspects of femicide in a judicial district of north eastern Italy. J. Forensic Leg. Med..

[26] Bonanni E., Maiese A., Gitto L., Falco P., Maiese A., Bolino G. (2014). Femicide in Italy: National scenario and presentation of four cases. Med. Leg. J..

[27] Zara G., Freilone F., Veggi S., Biondi E., Ceccarelli D., Gino S. (2019). The medicolegal, psycho-criminological, and epidemiological reality of intimate partner and non-intimate partner femicide in North-West Italy: Looking backwards to see forwards. Int. J. Leg. Med..

[28] Roesch E., Amin A., Gupta J., García-Moreno C. (2020). Violence against women during covid-19 pandemic restrictions. BMJ.

[29] Sánchez O.R., Vale D.B., Rodrigues L., Surita F.G. (2020). Violence against women during the COVID-19 pandemic: An integrative review. Int. J. Gynaecol. Obstet..

